# Data extraction for epidemiological research (DExtER): a novel tool for automated clinical epidemiology studies

**DOI:** 10.1007/s10654-020-00677-6

**Published:** 2020-08-27

**Authors:** Krishna Margadhamane Gokhale, Joht Singh Chandan, Konstantinos Toulis, Georgios Gkoutos, Peter Tino, Krishnarajah Nirantharakumar

**Affiliations:** 1 School of Computer Science, College of Engineering and Physical Sciences, University of Birmingham, Birmingham, B152TT UK; 2 Institute of Applied Health Research, College of Medical and Dental Sciences, University of Birmingham, Birmingham, B152TT UK; 3 Chair of Clinical Bioinformatics, Institute of Cancer and Genomic Sciences, College of Medical and Dental Sciences, University of Birmingham, Birmingham, B152TT UK; 4 Health Data Research UK, Birmingham, UK

**Keywords:** Epidemiology, Computer science, Extract, Transform, Load, Observational study, Research methods

## Abstract

The use of primary care electronic health records for research is abundant. The benefits gained from utilising such records lies in their size, longitudinal data collection and data quality. However, the use of such data to undertake high quality epidemiological studies, can lead to significant challenges particularly in dealing with misclassification, variation in coding and the significant effort required to pre-process the data in a meaningful format for statistical analysis. In this paper, we describe a methodology to aid with the extraction and processing of such databases, delivered by a novel software programme; the “Data extraction for epidemiological research” (DExtER). The basis of DExtER relies on principles of extract, transform and load processes. The tool initially provides the ability for the healthcare dataset to be extracted, then transformed in a format whereby data is normalised, converted and reformatted. DExtER has a user interface designed to obtain data extracts specific to each research question and observational study design. There are facilities to input the requirements for; eligible study period, definition of exposed and unexposed groups, outcome measures and important baseline covariates. To date the tool has been utilised and validated in a multitude of settings. There have been over 35 peer-reviewed publications using the tool, and DExtER has been implemented as a validated public health surveillance tool for obtaining accurate statistics on epidemiology of key morbidities. Future direction of this work will be the application of the framework to linked as well as international datasets and the development of standardised methods for conducting electronic pre-processing and extraction from datasets for research purposes.

**Electronic supplementary material:**

The online version of this article (10.1007/s10654-020-00677-6) contains supplementary material, which is available to authorized users.

## Background

Advancements in technology and healthcare systems has enabled large-scale collection of longitudinal electronic health records [1]. In the UK, there are many primary care databases (THIN, CPRD, QResearch and ResearchOne) of anonymised patient records [2, 3]. These datasets include information on demographics, practice registration related information, prescriptions, morbidity, lifestyle factors (height, weight, blood pressure, smoking and alcohol status) immunisation and laboratory test results [4]. The volume of data held in such datasets will continue to increase [5, 6].

Generally, the data within primary care databases are derived from healthcare software system used to manage patient’s clinical data [7, 8]. These systems are designed for the end-user experience of helping healthcare professionals to access and manage clinical data rather than for research purposes. As such, these datasets present several challenges related to missing data, variation in definitions for diagnoses and incomplete and inadequate capturing of secondary care information [9–11]. However, the strength of these databases lies in their size, breadth, representativeness of the population, long-term follow-up and sufficient data quality. Primary care databases offer great research potential related to drug safety and effectiveness research, identification of disease risk factors, generation of algorithms for identification of high risk patients, evaluation of public health policies and surveillance of diseases [4, 12–16]. In recent years, there is an increase in trend of using such routinely available data in healthcare research [3, 17].

In research which utilises primary care databases, data pre-processing and extraction are important steps of transforming the available raw data into a format suitable for statistical analysis. The process of extraction of primary care data for research is expensive due to time, effort and expertise required [11]. Factors such as; database size, database structure, range of available data, level of detail, complexity of study designs and study variables (such as exposure, outcome, inclusion or exclusion criterion and potential confounders) makes data extraction a complex process. It requires experts with considerable clinical, scientific and technical expertise to interrogate primary care databases [18]. A sound communication and documentation process is also important between researchers and data extraction experts to reduce human induced errors, and to minimize any biases that may occur in extracted data set because of miscommunication or difference in understanding. Data suppliers may offer extraction services for a fee, or researchers may collaborate with in-house specialists available to carry out extraction. There are no standard methodologies one can follow to extract data from primary care databases. An initial extraction may be based on patient restrictions, inclusion and exclusion criteria and multiple other extractions are performed to obtain additional variables. Experts use various software to manually compile an analysable data set for each study. This non-standard and non-automatic way of data extraction is labour-intensive; adds constraints on accuracy and reproducibility of results; and has limited methods to verify the validity and integrity of the datasets generated. Some corporate as well as University bodies have made considerable progress in trying to deliver solutions to manipulating electronic health record data. The rEHR R package, and The European Health Data & Evidence Network’s (EHDEN) Observational Health Data Sciences and Informatics (OHDSI) platform ATLAS are examples of software available to support researchers in extracting data taken from primary care [19, 20]. However, these packages and tools requires substantial user manipulation, statistical background and programming expertise which clinicians may lack and/or lacks certain features such as data extraction based on particular study designs and the ability to match cohorts. Aetion is an example of an alternate corporate provider of readily analysable datasets using longitudinal healthcare data, however, the mechanism and schema of their approach has not yet been published, therefore the validity and reproducibility of their approach is currently unclear [21].

In spite of best efforts, data extraction from primary care databases poses a number of issues; technical, human dependent, non-automatic, time consuming, need for data cleaning, handling very large data sets and complex logic with no room for verification and validation of the generated data. With advances in technology, there is clear scope in improving current methods of designing studies and extracting data. In particular, considering the importance of data in medical research, it is crucial to create automated methods to extract verifiable and valid datasets to expedite research and to avoid human induced errors.

In this paper, we introduce DExtER, an extract transform load (ETL) based software framework that enables automated clinical epidemiological studies (ACES), in a reproducible and verifiable way. This system potentially allows the stakeholders to extract high quality, patient-based data from primary care databases and hence enables a large range of research possible with electronic primary care data that could be translated to other healthcare databases.

## Methodology

### Extract, transform and load (ETL)

ETL [22] processes are backbones of data warehousing. Application of ETL is not new in medical research [23–26]. ETL performs three distinct steps; extraction of data from source system, transformation of data and loading of data into target system.

**‘Extraction’** is a simple process, in which depending on what stage the data is needed and what data is needed, tailored subset of data that is necessary for the subsequent transformation stage is extracted from specific parts of the data source. Extraction can be from multiple sources (multiple primary care databases or a combination of primary and secondary care databases) and from any form of technological infrastructure (or a combination of them) in which the data is being stored (e.g., RDBMS, NoSQL, spreadsheets and flat files).

During ‘**Transformation’** a number of operations can take place e.g., conversion, filtering, reformatting, application of a number of special-purpose business rules and aggregation. It is important to identify and note some of the schema level and instance level challenges that may occur during transformation [27]. For example, as the system allows multiple data sources, conflicts in naming can occur where same name might identify different things in the data sources. Different primary care databases may use different clinical coding systems (Read codes vs ICPC: International Classification of Primary Care). Value level problems may exist such as different date formats or HbA1c being expressed in  % as opposed to mmol/mol. Converting data structure and semantics of various databases into one common format, for example OMOP CMD [28], helps to solve some of these issues. The benefits of such unified data model has already been well researched [28–30].

Alternatively, several operations such as conversion, normalization and reformatting are accommodated in the transformation steps. Finally, in the ‘**Load’** step, the resulting transformed data gets pushed into the target systems or file formats.

### Flow of control and application to observational study design

Our system is based on observational (cohort, case–control, and cross-sectional) study designs. Although, the core principles of each observational studies are somewhat similar (observational in nature without introducing an intervention), they do have certain steps that do differ in terms of exposure definition, the need for controls and how they manage time. Hence the first step towards automating data extraction was to map out steps performed in these study designs. For example, a cohort study requires an exposed and unexposed group which are both followed up until a point of outcome or study end date.

**Fig. 1:**
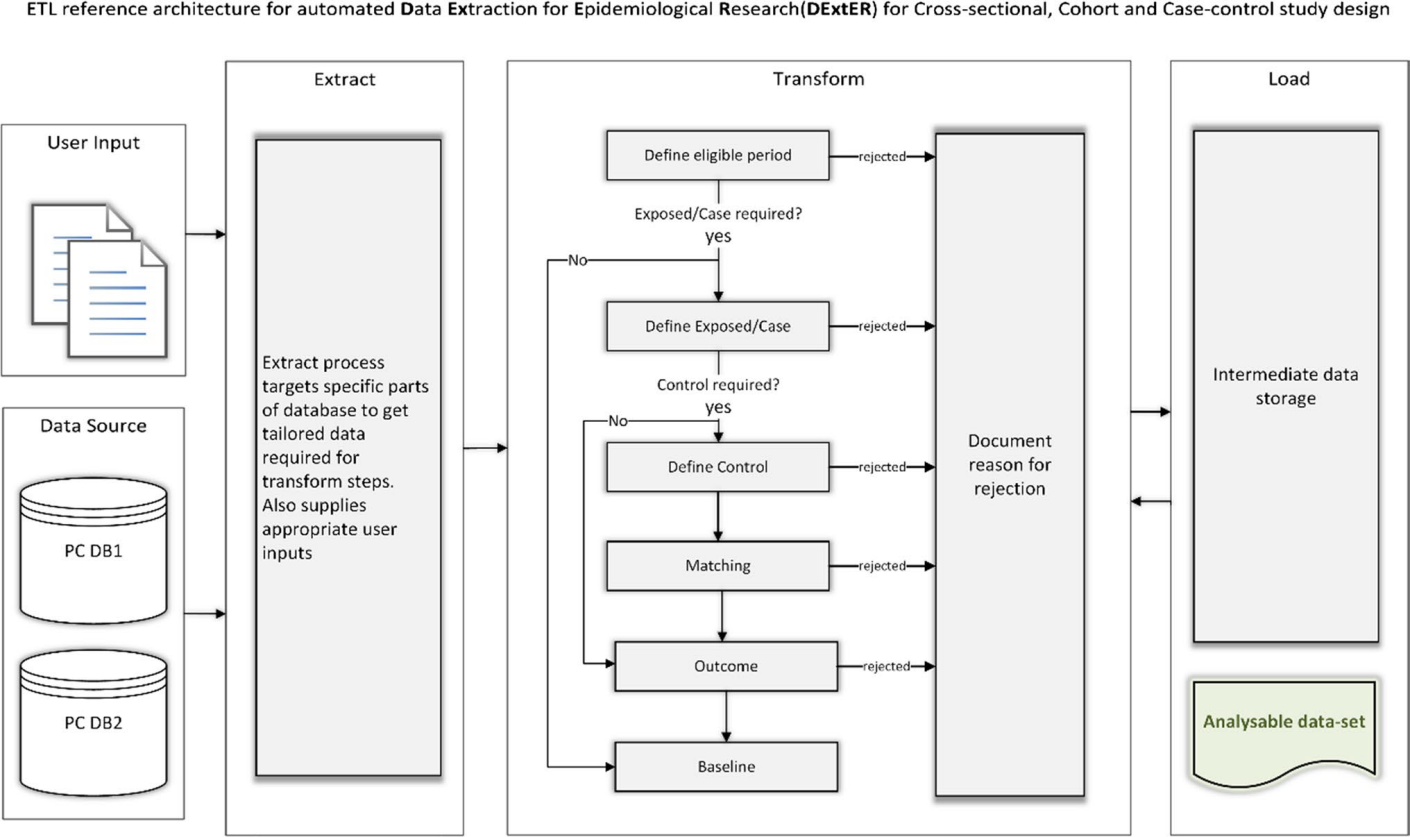
ETL workflow

Instead of creating a different ETL workflow for each study design, we identified common extraction steps and merged them while allowing necessary branches for steps unique to each design. This way we were able to create a single workflow model (Fig. 1) that is applicable for all the three study designs and understand the primary flow of control (ordered sequence of steps) that describes the route of data from the sources to the target, and intermediate transformations along the workflow. Defining this flow of control is essential for implementation and serves as the conceptual design of our novel model [31, 32] (i.e., the process of identifying the sources and the target systems, and determining the appropriate transformations).

Figure 1 also represents the ETL workflow and illustrates primary care database sources (PC DB1 and PC DB2), which could represent any electronic health record dataset. The raw data from each source propagates through different workflow stages of the system based on the study design and user input criteria. Depending on selected study design and input criteria, the extraction process may not involve all the transformation stages. For example, in a cross-sectional study, there is no control selection, outcome definition or matching required, and hence the extraction process would skip these stages. Before each transformation stage begins, an extract step will fetch tailored data from specific parts of the raw data sources based on the input criteria. After each stage of the transformation process, there are intermediate data stores that holds the transformed data until it propagates to the next stage. The intermediate data stores can be in-memory data structures (such as RAM) or can be stored in physical memory; the latter option are helpful in the case of a failure, where the whole process need not be started from the beginning. Ultimately, raw data is transformed into analysable datasets and loaded into the target system. An important process at the end of each transformation step is to document the reason why some patient records may be rejected and do not qualify for further processing. This documentation process is crucial and of immense importance, to ensure that data produced by the system is verifiable and valid, often a step which is not as easily achieved through other means. This process informs stakeholders of why patients were filtered out at various stages of data extraction and the reason for rejection. Another important feature of our model is its modular nature. Modularity of the system allows flexibility in terms of error handling, logic implementation, and provides ease to add new workflow stages (for example, a new workflow stage to clean the data set). A specific transformation stage of importance is added to encrypt the datasets using Advanced Encryption Standard (AES) 256-bit cipher as a data privacy step.

### Stages of study design input using DExtER

A pre-requisite for using the system to extract data is to come up with a well-defined set of inputs, ranging from; study period, study population, study variables (exposure, outcome and covariates), requirement of controls with matching criteria and the baseline characteristics with the outcome(s) of interest. In our implementation of the system, we have built a web-based HTML UI for users to provide these inputs, and we save the input in database and supply it to the system for data extraction based on a FCFS (First Come, First Serve) queue and hence provide multiuser facilities. The primary care database we used was ‘The health improvement network’ (THIN) database [33]. We have been able to test and validate the system on the Clinical Practice Research Datalink (CPRD) GOLD [34] and CPRD Aurum [35]. Our system is applicable to any observational healthcare database which is similar in structure and semantics to CPRD, THIN or observational medical outcomes partnership (OMOP) common data model (CDM).

#### Stage 1: Defining study eligible period

The first stage of the system is the process of defining eligible period for the study (Table 1). This eligibility period is composed of the patient start and end date, and it is in between this time duration in which all the events of interest (exposure, outcome) take place. In this step, we define the age and sex requirements of the group (e.g. in a study about pregnant woman we usually require only females of the age group 13–50 years) and apply data quality filters (e.g. adding days to keys dates of adequate computer usage) to improve the integrity of the raw data [36, 37].

**Table 1:** ETL stage 1: defining study eligible period

*ETL stage 1 user inputs*			
#	Variable name	Data type	Example
1	Study start date	Calendar date	15/01/1998
2	Study end date	Calendar date	15/12/2019
3	The number of days that should be added to key dates (Computerization/Acceptable Mortality Rate/Healthcare System) of the practice (optional)	Numeric	365
4	The number of days the patient should be registered in the practice before inclusion in the study (optional)	Numeric	365
5	Age at cohort entry	Numeric	25–84
6	Maximum allowed age at cohort exit	Numeric	115
7	Sex of the population	Categorical with following levels • Male • Female • Any	Any

*ETL stage 1 transformation logic*	
Transformation logic	
1. For each practice present in the database repeat the following • Add the number of days to key dates (input #3) • Practice start date = latest date of (key dates, Study Start Date) • Practice end date = earliest of (Study End date, Collection date) 2. For each patient record present in the eligible practice, repeat the following • Add the number of days to Registration date of the patient if input is supplied (input #4) • Patient start date = latest date of [Registration date, Practice Start Date, date the patient attains minimum age at cohort entry (input #5)] • Patient end date = earliest date of [Practice End date, Deregistration date, Death date, date the patient attains maximum age allowed at cohort exit (input #6)] • If patient is at least minimum age years old at patient start date, but not older than maximum age years old go to next step • If patient’s sex is same as what is supplied in input 7, go to next step • If patient start date is before practice end date and patient end date is after practice start date, store the patient record for the next stage else reject 3. Document reason for rejection	

#### Stage 2: Defining the exposed group in cohort design or cases in case–control design

The next stage is to define the exposed/case group as the system assigns each patient an index date based on single or multiple exposure (Table 2). In this step we employ a recursive descent parser (a mathematical tool used to determine if a sequence of symbols such as sentence is syntactically correct) [38], and a regular grammar (set of rules used to define the syntax for a particular language) [39] to successfully identify any number of exposures. If the study has more than one exposure, then we propose two modes of parsing namely strict parsing and loose parsing. The former is used when the order of occurrence of each exposure is relevant to the study (for example, in one of our study the exposure was diabetic patients who were prescribed a particular medication [40]), if otherwise, the latter is selected. If the study requires unexposed group/controls, patients who do not belong to the exposed group are labelled as potential unexposed group/controls. In supplementary 1 we discuss the proposed regular grammar.

**Table 2:** ETL stage 2: Defining the exposed group in cohort design or cases in case–control design

*Defining code entities*	
In electronic health records all diagnoses, symptoms, treatment, physical and laboratory measurements are coded into the system using some sort of clinical coding system, for example in Vision and EMIS systems, Read codes are used to record all diagnoses and symptoms. In HES records OCPC and ICD10 codes are used for the same purpose In this section we introduce a code entity which encapsulates clinical code that represents diagnoses, symptoms, treatment, physical or laboratory measurements etc. and some properties that describe it’s use and characteristic. The properties of Code Entities will change slightly depending on which ETL stage it is being used	

*Code entity for exposure*			
#	Variable	Data type	Example
1	Name	Text	Type2Diabetes
2	Criteria	Categorical with following levels • Inclusion criteria • Exclusion criteria	Inclusion criteria
3	Exposure type	*Categorical with following levels* For inclusion criteria • Incident only • Incident or prevalent • First record after cohort entry For exclusion criteria • Exclude if ever recorded • Exclude if recoded before index date	For inclusion • Incident only For exclusion • Exclude if ever recorded
4	Definition	Delimited text	Read code for Type 2 diabetes: C10F.11, C10F.00 ICD10 for Type 2 diabetes: E110

ETL stage 2 user inputs:			
#	Variable name	Data type	Example
1	Code entity	Code entity of exposure	Name: Type2 Diabetes Criteria: Inclusion criteria Exposure type: Incident only Definition: {C10F.11, C10F.00} Name: Metformin Criteria: Inclusion criteria Exposure type: Incident only Definition: {6.1.2.2}
2	Combination logic of the exposure	Text: formatted as regular grammar suggested	Type2Diabetes and Metformin
3	Parsing mode	Categorical with following levels • Strict • Loose	Strict
4	Exclude patients if these outcomes occur before index	Code entity (list)	Ischemic heart disease Stroke

*ETL stage 2 transformation logic*	
Transformation logic	
Repeat the following for each eligible patient record present from previous stage • For each code entity with inclusion criteria if the exposure type is ○ Incident only: find the earliest event of the code entity before patient end date and save details if found, if the event is before patient start date exclude patient and document reason for exclusion ○ Incident or prevalent: find the earliest event of the code entity before patient end date and save details if found ○ First record after cohort entry: find the earliest event of the code entity after the patient start date and before patient end date and save details if found • The parser in the system based on the combination logic supplied does the following each time it encounters a code entity of the patient ○ If all inclusion code entities are found in the patient go to next step, else if controls are required mark patient as ‘potential control’ else discard the patient and document reason ○ If the parsing mode is loose, latest event date among the code entities is set as patient’s index date ○ Else if the parsing mode is strict, If and only if the code entities have occurred in the same order as defined in the combination logic, set patient’s index date as date of latest entities’ event date else discard the patient and document reason for rejection • For each code entity with exclusion criteria if the exposure type is ○ Exclude if ever recorded: find the event described by the code entity, if the entity is found exclude patient and record documentation for rejection ○ Exclude if recorded before index date: find the event described by the code entity before patient’s index date, if the entity is found exclude patient and record documentation for rejection • Exclude patients if these outcomes occur before index ○ For each code entity in the list check if it occurs before index date, if the entity is found exclude patient and record documentation for rejection	

During implementation of the system we have trialled some advanced study designs such as whether the exposure considered is only incident patients compared to occasions where we have explored incidence and prevalent cases particularly where the exposure is rare [40–44]. Current work involves implementing pharmaco-epidemiolocal study designs such as new [45] and prevalent new-user designs [46].

During this stage it is also important to understand how to manage the outcome variables. If the study requires patients to be removed if an event of the outcome has occurred before the index date, then the workflow stage for determining outcome(s) is executed right after each time an index date is assigned to the patient and then patients are removed if the outcome has occurred before index date. If the study does not have any restriction on the patient based on the outcome the workflow stage for determining outcome(s) is executed before the last stage.

#### Stage 3 and 4: Defining the control group and matching

The control selection stage (stage 3) executes only if the controls are required for the study (Table 3). We note that unexposed/control group can still have exposure(s). Although the index date for controls depends on the clinical question and study design, in most studies the corresponding exposed patient’s index date is assigned to the control patient to mitigate immortality time bias [47].

**Table 3:** ETL Stage 3: Defining the control group

This stage is very similar to the previous stage in many ways. The he definition of the code entity remains the same as in the previous stage			
*ETL stage 3 user inputs*			
#	Variable name	Data type	Example
1	Code entity	Code Entity of exposure	Name: Type2Diabetes Criteria: Inclusion criteria Exposure type: Incident only Definition: {C10F.11, C10F.00}
2	Combination logic of the exposure	Text: formatted as regular grammar suggested	Type2Diabetes
3	Parsing mode	Categorical with following levels • Strict • Loose	Loose

*ETL stage 3 transformation logic*	
Transformation logic	
Repeat the following for each eligible patient record marked as ‘potential control’ from previous stage • For each code entity with inclusion criteria if the exposure type is ○ Incident only: find the earliest event of the code entity before patient end date and save details if found, if the event is before patient start date exclude patient and document reason for exclusion ○ Incident or prevalent: find the earliest event of the code entity before patient end date and save details if found ○ First record after cohort entry: find the earliest event of the code entity after the patient start date and before patient end date and save details if found • The parser in the system based on the combination logic supplied does the following each time it encounters a code entity of the patient ○ If all inclusion code entities are found in the patient go to next step, else discard the patient and document reason ○ If the parsing mode is loose, latest event date among the code entities is set as patient’s index date ○ Else if the parsing mode is strict, If and only if the code entities have occurred in the same order as defined in the combination logic, set patient’s index date as date of latest entities’ event date else discard the patient and document reason for rejection • For each code entity with exclusion criteria if the exposure type is ○ Exclude if ever recorded: find the event described by the code entity, if the entity is found exclude patient and record documentation for rejection	

The matching stage (stage 4) of the ETL workflow is employed where controls are required for the study and they need to be matched on specific parameters (Table 4). The matching criteria in each epidemiological study varies dependent on the study design. Parameters such as age, sex, and practice-based matching are common, some criteria such as matching disease duration (example diabetes duration), matching variable values (e.g. HbA1c or BMI) are also required at times. In our implementation of the system we have provided a range of matching criteria such as the number of unexposed/controls needed per exposed/case; should unexposed/control come from the same practice or from randomly selected practices; and what parameters they need to be matched on (e.g. age, gender and other variables described above).

**Table 4:** ETL Stage 4: Matching

In this step we match and assign controls to the exposed/case groups that we have defined in the previous two steps. In this stage we ignore the exposure type and criteria properties of the code entities			
*ETL stage 4 user inputs*			
#	Variable name	Data type	Example
1	Number of controls required for each exposed	Numeric	4
2	Plus, or minus how many years old can the control be compared to exposed	Numeric	1
3	Match on same sex	Categorical with following levels • Yes • Any • Opposite	Yes
4	Plus or minus how many days should we match on registration date	Numeric	365
5	Match on ethnicity	Categorical with following levels • Yes • No	No
6	Match on townsend score	Categorical with following levels • Yes • No	No
7	Match for exposure duration?	Categorical with following levels • Yes • No	Yes
8	Which exposure duration to match for and for how long (in days)?	Code entity, numeric	Type2Diabetes, 365
9	Match for conditions at baseline, if yes what to match for?	Code entity	Hypertension
10	Match for treatment at baseline, if yes what to match for?	Code entity	Aspirin
10	Match for physical measurements if yes with-in what time duration (in days) and to plus or minus what value?	Code entity, numeric, numeric	BMI, 735, ± 2
11	Match for laboratory results if yes, with-in what time duration (in days) and to plus or minus what value?	Code entity, numeric, numeric	HBA1C, 735, ± 2
12	Exclude patients if this outcome event occurs before index date	Code entity (list)	Stroke TIA Ischemic heart disease
13	Exclude patients if this event occurs before index date	Code entity (list)	Obstructive sleep apnoea

*ETL stage 4 transformation logic*	
Some of the steps described below may not be executed depending on the input supplied by the user. For example, if the user does not wish to match for Townsend then matching for Townsend step would be skipped from execution	
Transformation logic	
1. Randomise the list of exposed patients and the list of potential controls 2. After randomisation repeat the following for each exposed • Filter out all potential controls based on supplied sex matching criteria • Filter out all potential controls whose patient end date is before exposed/case’s index date or patient start date is after exposed/case’s index date • Calculate age as on exposed/case index date for all remaining controls. Filter out all potential controls if their they are too old or too young based on the given input criteria • Filter remaining potential controls based on given Townsend matching criteria • Filter remaining potential controls based on given Ethnicity matching criteria • Filter remaining potential controls based on given Exposure duration matching criteria • Filter remaining potential controls by removing everyone who are not on the same treatment (from the input list supplied) as that of exposed • Filter remaining potential controls by removing everyone who are not on the same treatment (from the input list supplied) as that of exposed • Remove all potential controls who are not on the same treatment (from the input list supplied) as that of exposed/case before exposed/case’s index date • Remove all potential controls who do not have the underlying conditions (from the input list supplied) as that of exposed/case before exposed/case’s index date • Filter remaining potential controls by removing everyone whose required physical measurement and/or laboratory results are not with-in the range specified and with-in the time scale specified as that of the exposed/case • Filter remaining potential controls by removing everyone who has a record of outcome and/or record of an event specified on or before the exposed/case’s index date 3. In the list of remaining controls randomly pick as much as number of controls required per exposed/case and assign them an index date which is the same as exposed/case and remove them from the main list of potential controls (for a without replacement control selection) 4. Assign each exposed/case and their controls a group id	

We have developed the UI so that users can enter appropriate inputs for each of them. The matching stage of the workflow works in two steps. In the initial step, for each exposed/case in the study we identify and mark a list of unexposed/controls who pass the matching criteria. In the next step, we randomly (to avoid any biases) select the required number of controls for the exposed/case from the initial list and mark them as group by assigning them a unique number. Remaining unexposed/controls are unmarked and are available as potential controls for the other exposed/cases.

#### Stage 5: Determining outcome and defining patient exit date

This process introduces a new variable called the patient exit date (Table 5), which is by default the patient end date unless the study has an outcome in which case the patient exit date is set to the date on which the outcome occurred. This stage is also responsible to extract any outcome required for the study for all eligible patients.

**Table 5:** ETL Stage 5: Determining outcome and defining patient exit date

In this stage we ignore the exposure type and criteria properties of the code entities *ETL stage 5 user inputs*			
#	Variable name	Data type	Example
1	Outcome	Code entity (list)	Ischemic heart disease Stroke Heart failure

*ETL stage 5 transformation logic*	
Transformation logic	
For each patient from the previous stage, repeat the following • Set value of patient exit date to that of patient end date • Look for first event of required outcome(s) before patient end date and after patient’s index date • Assign exit date as date of the first outcome for each patient	

#### Stage 6: Baseline variables and assembling an analysable dataset

The last stage of the workflow is extracting baseline variables that are required for the study (Table 6). This is a simple extract step where for each patient the required variables (e.g. BMI, glucose levels) are extracted from the raw data sources based on the input criteria (example if the latest value is required or if the earliest value is required). This stage also hosts a final Load step, which assembles all the study variables from corresponding intermediate data stores (previously extracted and transformed at different stages of the workflow) as the analysable dataset. We also propose encrypting the generated datasets to standards set by NHS to enforce data protection.

**Table 6:** ETL Stage 6: Baseline variables and assembling analysable dataset

In this stage we ignore the exposure type and criteria properties of the code entities *ETL stage 6 user inputs*			
#	Variable name	Data type	Example
1	Baseline characteristic	Code entity (list)	Smoking Townsend score Mortality Blood pressure Diabetes treatment

*ETL stage 6 transformation logic*	
Transformation logic	
For each patient from the previous stage, repeat the following • Extract the required baseline variable • Assemble the patient’s data from all previous stages ○ Study population ○ Exposed/case information ○ Control information ○ Any matching information ○ Baseline characteristics ○ Outcomes • Encrypt the data using cipher supplied by user and write data to file/database • Exit process	

At the end of the process, we provide the reason for rejection of the discarded patients. This process is executed each time a patient is discarded. Here we employ a map data structure that maps reason for rejection to number of patients discarded for that reason. When the system encounters a patient, who is to be discarded, first it looks in the map to see whether it already contains the (key) reason for rejection which triggered the process. If present, then the value against this reason is incremented by one and patient is discarded. If the reason is not already present, then it is newly entered in the map, and the corresponding value is set to 1. This way we will be able to record all the patients who are rejected and the exact reason behind why they were rejected. Optionally, we can also log details of the patient to manually verify the rejection.

### Implementation on site

We have implemented DExtER as a 3-tier web-based software system. We have built a website as the front end where stakeholders of the system can login and use the UI to submit their study design and data extraction requests which are stored in a database. The middleware of our system is the data extraction software written in Java. The middleware processes the data extraction requests on a first come, first serve basis and notifies users when the data extraction is complete. The backend of the system is Postgres RDBMS to store THIN database available to our institution.

### Data protection and privacy

Data protection and privacy policies are important pre-requisites for any successful tool. Therefore, the web interface we developed for this tool are line with the University of Birmingham data regulation guidance and works within the principles set out by the Information Commissioners Office [48]. Prior to any data extraction, the study design must have gone through ethical approval. Following which, in accordance with the protocol sent for ethics, a minimum dataset is extracted. A data extraction log (audit) is created each time an extraction is attempted. Following extraction, the dataset is encrypted to AES 256 using a password supplied to the user.

## Discussion

To date we have conducted a wide variety of epidemiological studies to both validate our tool and shed light on complex clinical questions. This research has been conducted as part of funded investigator led research, doctoral research and postgraduate taught course dissertations. The results of such studies have culminated in over 35 peer-reviewed publications in high impact factor general medical and specialist journals in the last 2 years, with more than 25 studies currently ongoing simultaneously. We highlight some of the studies, present comparable research elsewhere as a source of validity and discuss their clinical and public health importance below.

### Utility and validity of DExtER

A summary of validation of the tool can be seen in supplementary 2. In our first study utilising the DExtER tool we explored the association between Type 1 Diabetes and subsequent risk of developing epilepsy using a cohort study design [49]. Previous literature were mainly case studies or case control in nature [49–52]. One study explored the association in a cohort design but the study was from a low-middle income country, where there are other confounding factors that may have resulted in the observed association. [53] In our study we identified exposed patients in THIN database with a diagnosis of Type 1 Diabetes and matched them to four unexposed patients by age, gender and general practice location. After following both sets of patients for, on average, just over 5 years, we were able to identify how many patients in each group developed epilepsy. The incidence rate (IR) of the development of epilepsy in the unexposed group was 44 per 100,000 person years. This rate is similar to published literature, as a recent systematic review described the IR to be 50/100,000 person years in developed countries globally [54] This was compared to an IR in our exposed group of 132/100,000 person years, resulting in an overall adjusted Hazard ratio (HR) of 3.01 (95% CI 1.93–4.68). This threefold increased risk was similar to cohort study from Taiwan (HR 2.84; 95% CI 1.95–4.69) [53].

We have conducted similar retrospective cohort study designs to report on outcomes including for rare diseases such as Achalasia [15] and IgA vasculitis [41]. For IgA vasculitis we identified patients with the child onset and adult onset IgA vasculitis and identified following adjustment that these patients had an increased risk of developing hypertension (Child onset: HR 1.52; 95% CI 1.22–1.89; Adult onset: HR 1.42; 95% CI 1.19–1.70) and chronic kidney disease (Child: HR 1.89; 95% CI 1.16–3.07; Adult: HR 1.54; 95% CI 1.23–1.93) [41]. With a similar study design, we identified that the diagnosis of the oesophageal condition achalasia was strongly associated with the development of oesophageal cancer and lower respiratory tract infections [15]. The findings in both of these studies are useful to clinicians as we can shed light on novel associations which are important to consider in clinical management and long-term surveillance.

These two manuscripts also highlight another application of the DExtER tool, which we have since utilised. We were also able to study yearly incidence and prevalence of these conditions using yearly cohort and cross-sectional study designs respectively. We noted that the incidence of IgA vasculitis was stable but the documented prevalence in the general population was increasing. In our Achalasia study, we were able to compare the documented IR from THIN data to that obtained from the Hospital Episodes Statistics. They were broadly similar (HES was 1.99 (95% CI 1.87–2.11) and 1.53 (95% CI 1.42–1.64) per 100 000 person-years in THIN), with observed difference potentially attributed to the differing population structure and incomplete recording of the condition in primary are settings. One of our publications on atrial fibrillation prevalence trends from 2000 to 2016 was extracted by a senior data scientist in the usual conventional manner by manually writing a programme using STATA software [55]. We compared the prevalence calculated by the data scientist to that obtained using DExtER, in the same year but using the latest version of the THIN database. Both were identical, for example both showed a prevalence of 3.3% in adults aged 35 years and older in 2016.

### DExtER as a public health surveillance tool

The scope and use of automated cohort and cross-sectional designs has the potential of being an extremely important assert in Public Health settings and drug safety surveillance centres. Working with colleagues in Public Health England (PHE) we have compared the prevalence the tool generates to that reported in Quality Outcome Framework and again we found that they were similar to prevalence observed from other UK data sources. We have now implemented the tool in PHE for generating key incidence and prevalence figures that will aid with service planning and resource allocation, and for further independent evaluation. As part of the agreement with PHE, they will be independently validating the use of the tool. The tool can also be used for surveillance of beneficial and adverse effects of medications, early on after introduction of new therapeutic agents and over a long period of time for outcomes such as cancer and cardiovascular events. For example we were able to explore whether benefits from medications demonstrated in clinical trials (such as the Empagliflozin EMPA-REG trial [56]) can be replicated in real-world settings. Sodium-glucose transport protein 2 inhibitors were found to decrease mortality in patients with previous cardiovascular disease in this trial. We were able to explore this in THIN database and found that patients who were given SGLT2 Inhibitors were significantly less likely to die of any cause irrespective of baseline CVD status (adjusted IRR 0.50; 95% CI 0.33–0.75) [14]. This specific finding was replicated by another large real world evidence study (CVD-REAL HR 0.49; 95% CI 0.48–0.60 [57]).

### Future directions, benefits, limitations and ethical considerations

We are now progressing the work towards automation for complex study designs through a work programme named Automated Clinical Epidemiology Studies (ACES), partly funded by Health Data Research (HDR) UK through a fellowship [58]. Additionally, through HDR UK, our team was involved in two successful digital innovation hubs (INSIGHT and PIONEER) [59]. As part of the INSIGHT hub funding, the tool is now also being adapted to include bespoke eye hospital data and provide further learning opportunities into the flexibility, benefits and limitations of the tool particularly for datasets not configured for use in observational research.

As part of the HDR fellowship work, we will apply the framework for pharmaco-epidemiology study designs, linked primary–secondary care databases and for databases with linked mothers and babies. How each of these can be incorporated into DExtER depends on what information should be extracted from them and can vary drastically depending on the research question. The important thing to note is that the modular nature of the tool allows the addition of new stages containing specific rules and complex scenarios to its current set to facilitate such datasets. For example, the tool has the capacity to conduct a study involving primary care and hospital episode statistics where a researcher maybe interested at looking readmissions to the hospital but may want to look in the primary care for the baseline variables. In such scenarios it is possible to add a new modular stage with bespoke UI to DExtER to facilitate this study design. We will also apply our tool to databases in other settings and countries, resulting in a global community who can collaborate and generate reproducible research across several databases. For example, we have evaluated its ability in the RNH (Registration Network for General Practitioners) database in Netherlands and found it to work seamlessly. The main benefits of the system are it provides researchers fast, efficient and reliable data extraction capacity. It eliminates the IT expertise required to extract datasets manually. The proposed ETL based architecture works as a standard to extract data for epidemiological studies and extracting data in this automated and standard way highly promotes reproducible research which is hard in epidemiology [60]. The documentation why patient records were discarded establishes data integrity, credibility and renders the dataset valid and verifiable. The tool has its limitation such as not being able to cater for all possible different subtle designs an epidemiologist may consider eliciting an association or methodologies to reduce biases in specific contexts. The tool can only be used with sound knowledge of epidemiological principles, otherwise may result in numerous spurious and potentially incorrect findings. The ability to conduct studies within hours could result in publication biases where researchers may choose to undertake studies with a prior knowledge of the likely outcome or chose to ignore pursuing studies with negative outcomes [61]. To avoid this, the team will aim to conduct workshops involving key stakeholders to build an ethical framework that mitigates these unintended consequences of ACES. To facilitate global research, currently the tool can be made available for research at academic institutions anywhere in the world, subject to a negotiated contractual licence with the University of Birmingham (contact the corresponding author for further details).

## Conclusion

In the recent years, there is an increasing trend in the use of routinely available data in the field of epidemiology. Primary care databases supply researchers with large amounts of medical data and the potential to answer several different research questions using various study designs. However, nonstandard and manual data extraction from primary care databases is complex, labour intensive and time-consuming process. Currently existing solutions such as the rEHR package and EHDEN’s ATLAS [19, 20] attempt to overcome the need for manual extraction, but these options still require substantial programming skills (need for expertise and prone to human error) and are limited in its applicability to various study designs and matching options. Whereas in this paper, we have been able to present an ETL based framework (DExtER), a tool used to automate the process of data extraction for epidemiological research based on study designs which can utilise any longitudinal primary care electronic record source. Corporate solutions which can provide a similar service to DExtER, [21] are yet to publish the developed algorithms/programs, and hence they are limited in the validity of their techniques and reliability in the datasets generated for research. Whereas, DExtER provides a non-invasive solution to generate quality datasets in an analysable format through a process that can be verified and reproducible.

We anticipate this new architecture will expedite and reduce the costs of epidemiological and health services research by reducing the gap between medical researchers and electronic patient records. As a part of the future work, we want to develop concrete standards for each step in the data extraction process and work towards developing automated analytics with the vision to create an automated research pipeline for epidemiological studies.

## Electronic supplementary material

Below is the link to the electronic supplementary material.

Supplementary material 1 (DOCX 14 kb)

Supplementary material 2 (DOCX 20 kb)
