# Supplementary material for: Data extraction for epidemiological research (DExtER): a novel tool for automated clinical epidemiology studies
Source: Eur J Epidemiol. 2020 Aug 27;36(2):165–78. doi: 10.1007/s10654-020-00677-6 (PMC7987616; doi:10.1007/s10654-020-00677-6)
Supplement: Supplementary file 1 — Supplementary material 1 (DOCX 14 kb) [file 10654_2020_677_MOESM1_ESM.docx]

# Supplementary 1: Grammar process

The regular grammar involved in the parsing process can be defined as follows, let us say “G” represents the regular grammar. Then,

**G = (V, T, P, S)** where,

**V** is a finite set of a non-terminal symbols. Each entry represents a different type of phrase or clause in the sentence and defines a sub-language of the regular language defined by G. and V is defined as the following.

V = (Exp, Term, Fact)

**T** is a finite set of terminals, which make up the actual content of the language that can be produced using the proposed regular grammar. The set of terminals in **T** is the alphabet of the language defined by the grammar **G**. Please note that the <exposure> is just a placeholder for the actual exposure code that stakeholder supplies.

T= (“and”, “or”, “(”, “)”, “<exposure>”)

**S** is the start symbol used to represent the whole sentence.

**P** is also a finite set, which corresponds to the rules of the grammar. It is only by applying these rules a correct sentence can be produced. They replace nonterminal symbols (on the left side of the production) in a string with other nonterminal or terminal symbols (on the right side of the production). The following are the production rules of the grammar that is used in the system.

***S → Exp***

***Exp → Term Fact***

***Fact → and Term | or Term | ϵ***

***Term → ( Exp ) | < exposure>***
