# Supplementary material for: Data extraction for epidemiological research (DExtER): a novel tool for automated clinical epidemiology studies
Source: Eur J Epidemiol. 2020 Aug 27;36(2):165–78. doi: 10.1007/s10654-020-00677-6 (PMC7987616; doi:10.1007/s10654-020-00677-6)
Supplement: Supplementary file 2 — Supplementary material 2 (DOCX 20 kb) [file 10654_2020_677_MOESM2_ESM.docx]

# Supplementary table 2: Summary of validation of DExtER

| **Our study design** | **Dataset characteristics** | **Outcome measure** | **Comparison method** | **Comparative publication design** | **Comparative Dataset characteristics** | **Comparative outcome measure** |
| --- | --- | --- | --- | --- | --- | --- |
| The incidence rate of epilepsy in the UK general population [4] | UK participants (n=19,688) derived from THIN | Incidence rate: 44 per 100,000 person-years | Compared to systematic review data | The incidence rate of epilepsy in developed countries [5], the UK data is derived from Macdonald et al. [6] | UK participants (n=100,230) derived from the General Practice Linkage Scheme where GP data is linked to hospital specialty data | Incidence rate: 46 per 100,000 person-years |
| The hazard ratio (HR) of developing epilepsy in patients with Type 1 diabetes [4] | UK participants derived from THIN (case: n=4,922; control n=19,688) | Adjusted HR:  3.01 (95% CI 1.93-4.68) | Compared to a similarly designed cohort study | Taiwanese cohort study examining the risk of developing epilepsy in those with Type 1 diabetes 51] | Taiwanese participants from the Taiwan National Health Insurance Research Database (case: 2,568; control: 25,680) | Adjusted HR:  2.84 (95% CI 1.95-4.69) |
| The incidence rate of achalasia in the UK general population [7] | All eligible UK participants derived from THIN between 2006-2016 | Incidence rate:  1.53 (1.42 to 1.64) per 100,000 person-years | Comparison to figures from another national dataset | Calculation of the incidence rate of achalasia diagnosed in secondary care | All eligible UK participants derived from Hospital Episode statistics between 2006-2016 [7] | Incidence rate:  1.99 (95% CI 1.87 to 2.11)100,000 person-years |
| Examining the incidence rate ratio (IRR) of mortality associated with sodium-glucose transport protein 2 (SGLT-2) inhibitors in patients with cardiovascular disease (CVD) [8] | UK participants derived from THIN (case: n=4,444; control n=17,680) | Adjusted IRR: 0.50 (95% CI 0.33-0.75) | Comparison to another global real-world evidence study (CVD-REAL) | The hazard ratio associating the mortality risk in those with pre-existing CVD taking SGLT-2 inhibitors [9] | Participants taken from the CVD REAL study (case n=107,811; control n=107,811) | Hazard ratio: 0.49; 95% CI 0.48-0.60 |
| Examining the prevalence of atrial fibrillation (AF) in those aged over 35 years in 2016 [10] | UK participants derived from THIN | Prevalence: 3.3% in those aged over 35 years | Comparison to manual data extraction from the same dataset | Using manual data extraction methods to calculate the prevalence of AF in those aged over 35 years in 2016 | UK participants derived from THIN | Prevalence: 3.3% in those aged over 35 years |
| Examining the age-sex standardised prevalence of comorbidities of all those in THIN | UK participants derived from THIN | Atrial fibrillation: 1.75%  Stroke: 1.79% | Compared to published quality and outcomes framework estimates | Data taken from Public Health England when examining comorbidities | Data taken from Public Health England national statistics | Atrial fibrillation: 1.71%  Stroke: 1.74% |
